# Acetate and combinations of short-chain fatty acids increase oxidative phenotype and contribute to muscle fiber type shift in myotubes

**DOI:** 10.3389/fphys.2026.1757576

**Published:** 2026-03-25

**Authors:** Britt M.J. Otten, Mireille M. J. P. E. Sthijns, Harry R. Gosker, Marco C. J. M. Kelders, Freddy J. Troost

**Affiliations:** 1 Department of Human Biology, NUTRIM, Institute of Nutrition and Translational Research in Metabolism, Faculty of Health, Medicine and Life Sciences, Maastricht University, Maastricht, Netherlands; 2 Food Innovation and Health, Centre for Healthy Eating and Food Innovation (HEFI), Maastricht University, Venlo, Netherlands; 3 Department of Respiratory Medicine, Institute of Nutrition and Translational Research in Metabolism (NUTRIM), Faculty of Health, Medicine and Life Sciences, Maastricht University, Medical Centre+, Maastricht, Netherlands

**Keywords:** C2C12 myotubes, SCFAs, oxidative fiber type, Pgc1α, *Myh7*

## Abstract

**Introduction:**

Intestinal microbial fermentation produces short-chain fatty acids (SCFAs) that signal from the large intestine to skeletal muscle. Skeletal muscle exhibits phenotypic plasticity, with fiber-type composition shifting between oxidative (slow-twitch) and glycolytic (fast-twitch) states in response to metabolic and environmental cues. While SCFAs have been implicated in modulating metabolism, their role in skeletal muscle fiber-type regulation remains poorly defined. This study aimed to investigate whether acetate and SCFA mixtures (acetate, propionate, and butyrate) can promote a shift towards an oxidative skeletal fiber type in skeletal muscle cells.

**Methods:**

Cultured C2C12 myotubes were exposed to SCFAs, and fiber type-specific gene and protein expression was assessed. Exposure: Exposure to ≥0.5 mM of the SCFA mixture for 8 h increased *Pgc1α* and *Tfam* gene expression compared to 10 mM acetate. This effect persisted after 24h exclusively in the SCFA mixture. *Pparα* gene expression at 8h was increased by ≥5 mM acetate and 10 mM SCFA mixture. *Myh7* gene expression increased after 24h with ≥0.5 mM SCFA mixture and ≥5 mM acetate. After 48h, ≥0.5 mM acetate increased myosin heavy chain (MyHC) I staining, whereas 10 mM SCFA mixture reduced MyHC II. By 72h, the mixture further enhanced MyHC I and sustained MyHC II reduction.

**Conclusion:**

This study shows that both acetate and SCFA combinations shift muscle myotubes towards oxidative fiber phenotype, with the mixture demonstrating a more pronounced effect at lower concentrations. This supports a role for gut-derived metabolites in muscle adaptation and demonstrates that SCFAs promote a shift toward an oxidative fiber type.

## 1 Introduction

Skeletal muscle is a dynamic tissue that adapts to various physiological demands and environmental cues (Imperatrice et al., 2022). It is able to alter its fiber-type composition in response to different stimuli, thereby optimizing its functional properties (Egan and Zierath, 2013). Muscle fibers can be categorized into three main types: slow-twitch (Type I, expressing myosin heavy chain (*Myh) 7)*, fast-twitch oxidative (Type IIa, expressing *Myh2*), and fast-twitch glycolytic (Type IIb or IIx, expressing *Myh4* and *Myh1*, respectively), each possessing distinct metabolic and contractile characteristics (Bourdeau Julien et al., 2018). Oxidative fibers are rich in myoglobin and have a high aerobic metabolism capacity, which is primarily advantageous for prolonged endurance-based activities. In contrast, glycolytic fibers, with fewer mitochondria, rely predominantly on anaerobic metabolism, providing bursts of energy for rapid, powerful contractions, but tend to fatigue more quickly. Mitochondria play a central role in determining a muscle fiber’s oxidative or glycolytic characteristics (Mishra et al., 2015). The regulation of muscle fiber-type composition is a complex process and is driven by multiple factors, including the regulation of gene expression and metabolic adaptations (Egan and Zierath, 2013).

The gastrointestinal tract is a key regulator of skeletal muscle function (Lahiri et al., 2019; Frampton et al., 2020; Chew et al., 2023). It provides substrates for energy production due to its capacity to digest and absorb food and food-derived metabolites and generates signals to skeletal muscle through a diversity of mechanisms, including the immune system, hormones, and microbial metabolites. This intricate interplay between the gut and skeletal muscle is often referred to as the gut–muscle axis. Previous research has shown that modulation of the gut microbiome can induce increases in endurance performance, both in murine models and in humans (Fernández et al., 2021; Furber et al., 2022; Morita et al., 2023). It is mediated in part by short-chain fatty acids (SCFAs) which are produced during microbial fermentation of primarily undigestible substrates (Chew et al., 2023).

Long-chain fatty acids (LCFAs), such as polyunsaturated fatty acids (PUFAs) and palmitate, have been shown to regulate muscle fiber type composition via PPAR signaling and mitochondrial biogenesis (de Wilde et al., 2008; Mizunoya et al., 2013; Watanabe et al., 2020; da Paixao et al., 2021). However, the role of short-chain fatty acids (SCFAs) in muscle fiber plasticity remains less well understood. SCFAs, such as acetate, propionate, and butyrate, have gained attention for their potential roles in influencing skeletal muscle (Frampton et al., 2020; Sales and Reimer, 2023). In healthy individuals, the molar ratio of acetate to propionate to butyrate in peripheral blood is around 80:10:10 (Ktsoyan et al., 2016). However, in a previous study, we explored variations in this ratio, such as 60:20:20, which was more potent to increase glucose uptake in C2C12 myotubes compared with 80:10:10 (Otten et al., 2023). Since SCFA concentrations in circulation can fluctuate based on dietary intake and gut microbial composition, it is relevant to investigate how different SCFA ratios impact skeletal muscle metabolism. The 60:20:20 ratio was selected as it elicited a stronger metabolic response, suggesting a potential for greater physiological relevance in modulating muscle function.

SCFAs engage with specific receptors such as G-protein-coupled receptors (GPRs) located on the cell surface of skeletal muscle cells (Giron et al., 2022). These receptors serve as gateways, transmitting SCFA-initiated signals into the cell. Once this signal enters the cell, intricate intracellular signaling cascades, involving peroxisome proliferator-activated receptor gamma coactivator 1-alpha (PGC1α), are initiated (Giron et al., 2022). PGC1α, a central regulator in skeletal muscle cells, interacts with peroxisome proliferator-activated receptors (PPARs) and nuclear respiratory factors (Nrf)1 and Nrf2a to stimulate mitochondrial gene transcription, thereby promoting the formation of new mitochondria (Kang and Li Ji, 2012; Abu Shelbayeh et al., 2023). This cascade of events, including the promotion of mitochondrial transcription factor A (TFAM) by PGC1α, leads to enhanced mitochondrial DNA (mtDNA) replication and transcription (Kang and Li Ji, 2012). Moreover, SCFAs engage with PPARs, particularly PPARα, which suggests a potential link between SCFAs and lipid utilization, known to be an essential aspect of endurance exercise capacity (Frampton et al., 2020). Overexpressing active *Pparδ* in mice was shown to increase type I myofibers and improved exercise capacity, whereas *Pparδ* gene deletion resulted in reduced expression of slow-twitch muscle genes (Wang et al., 2004). It was previously demonstrated that exposing C2C12 myotubes to a combination of SCFAs increased *Pgc1α* and *Tfam* gene expression (Lahiri et al., 2019). Acetic acid was found to activate AMP-activated protein kinase (AMPK) in myoblasts, leading to enhanced glucose and fatty acid uptake and reduced triglyceride accumulation, thereby suggesting a role for acetate in improving glucose and lipid metabolism (Lin et al., 2002).

While skeletal muscle’s ability to adapt its fiber type composition to changes in physical activity is well recognized (Yan et al., 2011; Plotkin et al., 2021), the role of SCFAs on the regulation of muscle fiber type composition is not clear. The activation of Pgc1α, induced by SCFAs, may stimulate the transition of muscle myotubes toward a more oxidative phenotype. This study aims to investigate the impact of SCFAs on muscle fiber composition and metabolic adaptations. Therefore, it was questioned whether a combination of SCFAs in a 60:20:20 ratio can induce a shift toward a more oxidative muscle fiber phenotype in C2C12 myotubes compared with acetate alone. To achieve this, we assessed key markers of mitochondrial biogenesis and function, including the gene expression levels of *Pgc1α*, *Tfam*, *Nrf1*, and *Nrf2a*. Additionally, metabolic adaptations were studied by investigating the gene expression of *Pparα* and *Ppar*δ, which play pivotal roles in fatty acid oxidation. Furthermore, muscle fiber type was studied by assessing the expression of myosin heavy chain isoforms genes *Myh7*, *Myh2*, *Myh1*, and *Myh4* and the protein expression of slow-twitch (MyHC I) and fast-twitch (MyHC II) myosin heavy chains.

## 2 Methods

### 2.1 Chemicals

Acetic acid, propionic acid, butyric acid, paraformaldehyde, and β-mercaptoethanol were purchased from Sigma-Aldrich (Saint Louis, Missouri, USA) as well as bovine serum albumin fatty-acid free (BSA), 4-(2-hydroxyethyl)-1-piperazineethanesulfonic acid (HEPES), Triton X-100 (TX-100), primary antibodies MyHC I (Cat# M8421) and MyHC II (Cat# M4276), and 4′,6-diamidino-2-phenylindole (DAPI). Primers, ethanol, and secondary antibody labeled with Alexa Fluor 488 were purchased from Thermo Fisher Scientific (CA, USA). The RNeasy mini kit was purchased from Qiagen (Hilden, Germany). The iScript kit as well as SYBR Green were purchased from Bio-Rad (Bio-Rad, CA, United States).

### 2.2 Cell culture and treatments

**Figure 1:**
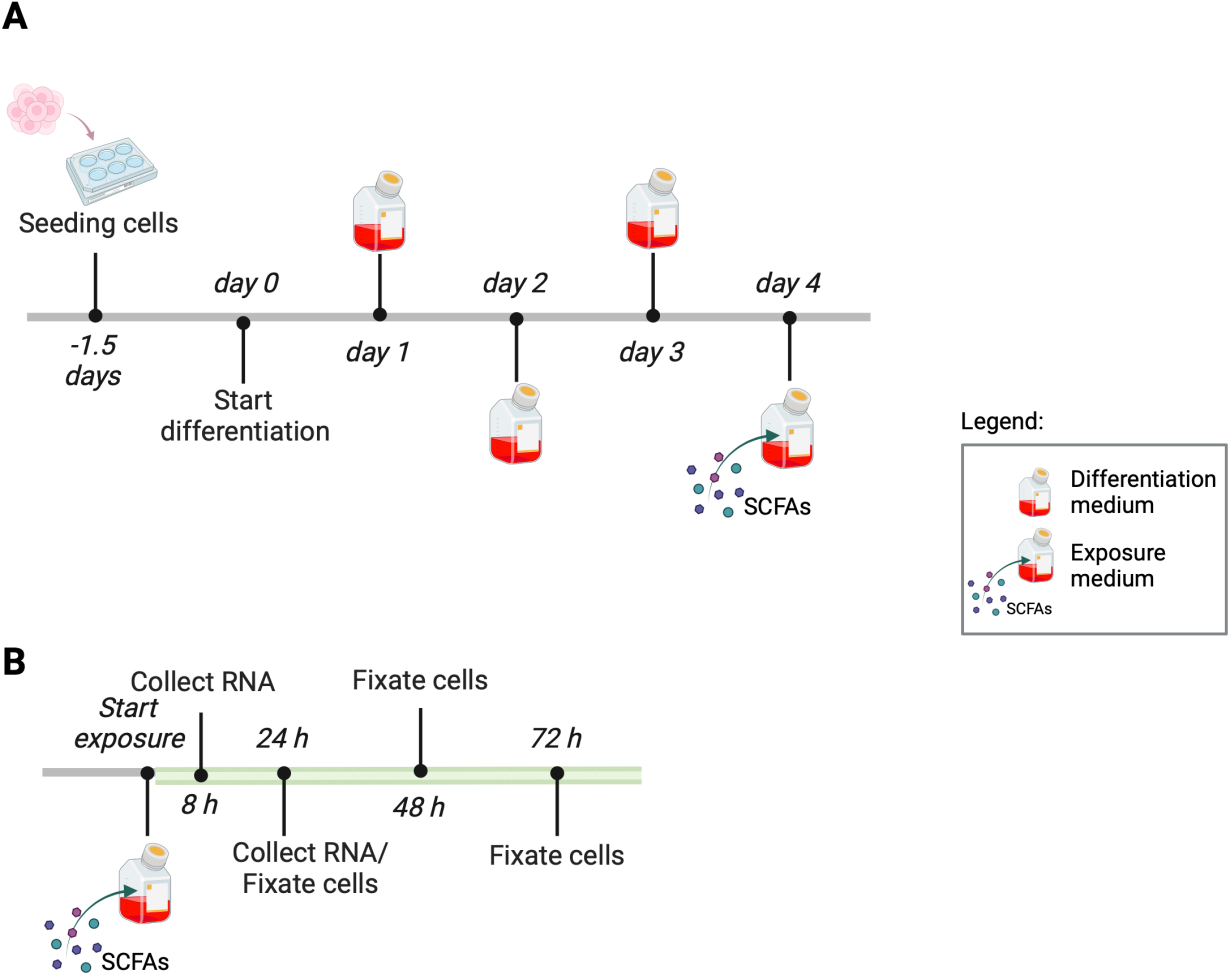
Experimental timeline. Timeline of the differentiation process of C2C12 cells **(A)**. Timeline of the experimental treatment of C2C12 myotubes **(B)**.

A C2C12 murine myoblast cell line (ATCC; CRL-1772; Manassas, VA, USA), at passages 9-16, was used, and growth medium (GM), composed of low-glucose Dulbecco’s Modified Eagle medium (DMEM, Gibco, Carlsbad, CA, USA) supplemented with 9% (v/v) fetal bovine serum (FBS, Gibco) and 1% (v/v) antibiotics (100 µg/mL penicillin, 100 µg/mL streptomycin, Gibco), was added to the cells. A humidified atmosphere with 5% CO_2_ at 37°C was applied to culture the cells until 70%-80% confluency. Depending on the experiment, 6-well or 24-well culture plates (Greiner Bio-One, Frickenhausen, Germany) coated with Matrigel (Corning Life Sciences, Corning, NY, USA) were used to culture the C2C12 myoblasts. The seeding density was maintained at 1*10^4^ cells·cm^−2^, and the cells were grown in GM for 1.5 days until reaching ±95% confluency (**Figure 1A**). Subsequently, a washing step was performed using Dulbecco’s phosphate-buffered saline (PBS, Gibco). Then, cells were incubated in differentiation medium (DM), which was composed of high-glucose DMEM with 1% (v/v) heat-inactivated FBS (hiFBS, Gibco) and 2.5% (v/v) HEPES, for three consecutive days. DM was renewed daily. Following 4 days of differentiation, the cells were cultured for 8, 24, 48, or 72h in exposure medium composed of 3% (w/v) BSA in high-glucose DMEM (**Figure 1B**). The exposure medium contained either acetate or a mixture of SCFAs in the following ratio: 60% acetate, 20% propionate, and 20% butyrate at concentrations of 0.5, 5, or 10 mM (**Table 1**). SCFA concentrations were selected based on previous work in C2C12 myotubes (Watanabe et al., 2020), indicating that high concentrations, e.g., 20 mM, induced cytotoxicity, whereas lower concentrations elicited biological responses without affecting viability.

**Table 1:** Composition of mixture of SCFAs.

		Acetate		Propionate		Butyrate	
	Concentration of total SCFA (mM)	Relative amount (%)	Concentration (mM)	Relative amount (%)	Concentration (mM)	Relative amount (%)	Concentration (mM)
Mixture	0.5	60	0.3	20	0.1	20	0.1
	5	60	3	20	1	20	1
	10	60	6	20	2	20	2

### 2.3 RNA extraction and qPCR analysis

Total RNA was extracted from C2C12 myotubes using the RNeasy Mini kit (QIAGEN) according to the manufacturer’s instructions (**Figure 1B**). The RNA was stored at −80°C until further processing. As a negative control for *Myh* expression, undifferentiated unexposed myoblasts were used. RNA quantity was assessed by a Synergy HTX Multimode Reader and Take3 Microvolume Plate and analyzed by Gen5 software (BioTek, Vermont, United States). The iScript cDNA synthesis kit was used to convert 500 ng RNA to complementary DNA (cDNA). The PCR tubes were placed in the CFX Connect Real-Time PCR Detection System (Bio-Rad, CA, United States) and incubated for 5 min at 25°C, 20 min at 46°C, 5 min at 85°C, and 7 min at 4°C. After completion, cDNA was stored at −20°C. Primers were designed using the Ensembl genome browser for template sequences and Primer3Plus for generation of primer sequences (annealing temperature 63°C) (Untergasser et al., 2007; Aken et al., 2016). Primers validated for their amplification efficiency were used at a concentration of 4.5 µM (**Table 2**). Quantitative PCR (qPCR) was done in a 15-µL reaction, combining 5 µL of diluted cDNA (1:50) with 10 µL PCR master mix containing 7.5 µL iQ SYBR Green Supermix, 0.45 µL of each sense and antisense primer, and 1.6 µL RNA free water. The qPCR was conducted on a Real-Time PCR Detection System in a 96-well PCR plate. Samples were incubated for 3 min at 95°C, and the thermocycling was 12 s at 95°C and 30 s at 55°C for 38 cycles. Relative RNA expression levels were calculated using the Pfaffl method and normalized to the expression of three housekeeping genes (GAPDH, RPL13A, and YWHAZ) (Pfaffl, 2001). Samples were expressed as fold change compared with controls without exposure to SCFAs.

**Table 2:** List of sequences of PCR primers.

Gene name	Sense primer sequence (5′ to 3′)	Antisense primer sequence (5′ to 3′)
Glyceraldehyde-3-phosphate dehydrogenase (GAPDH)	CAACTCACTCAAGATTGTCAGCAA	TGGCAGTGATGGCATGGA
Myosin heavy chain I (Myh1)	CAGATCGGGAGAATCAGTCCAT	AGCAAAATATTGGATGACCCTCTTA
Myosin heavy chain 2A (Myh2A)	GCCCCGCCCCACATC	GGATTGACTGATTCTCCCTGTCTGT
Myosin heavy chain 2B (Myh2B)	AGGGCGGCGGTGGAA	TGGGAATGAGGCATCTGACAA
Myosin heavy chain 2X (Myh2X)	CCTAGCCAAAGCCGTCTATGAG	GGTGGTTGAAGAACTGTTGCAGTT
Nuclear respiratory factor 1 (Nrf1)	AGCCACATTGGCTGATGCTT	GGTCATTTCACCGCCCTGTA
Nuclear respiratory factor 2 alpha (Nrf2a)	TGCTGCACTGGAAGGCTACA	TTACCCAAACCACCCAATGC
Peroxisome proliferative activated receptor, gamma, coactivator 1 alpha (Pgc1α)	CAAGAGCAAGTATGACTCTCTGG	CCTCAGCCTGGGAACACGTTAC
Peroxisome proliferator activator receptor alpha (Pparα)	ACTACGGAGTTCACGCATGTG	TTGTCGTACACCAGCTTCAGC
Peroxisome proliferator activator receptor delta (Pparδ)	AGGCCCGGAGCATCCTCA	TGGATGACAAAGGGTGCGTTG
Ribosomal Protein L13a (RLP13a)	TGGATATGCCCTTGACTATAATGAGTAC	AGGACTCCTCGTATTTGCAGATTC
Transcription factor A, mitochondrial (Tfam)	CCGGCAGAGACGGTTAAAAA	TCATCCTTTGCCTCCTGGAA
Tyrosine 3-monooxygenase/tryptophan 5-monooxygenase activation protein zeta (YWHAZ)	TGCTGGTGATGACAAGAAAGGAA	AACACAGAGAAGTTGAGGGCCA

### 2.4 Immunofluorescence staining

C2C12 myoblasts were seeded on 24-well plates and differentiated for 4 days. After differentiation, myotubes were exposed to single or mixed SCFAs in different concentrations for 48 or 72 h (**Figure 1B**). Based on previous studies demonstrating that changes in myosin heavy chain protein expression in C2C12 myotubes typically become detectable after prolonged exposure periods rather than at early time points, MyHC I and II protein levels were assessed at 48 and 72 h (Kuninger et al., 2006; Brown et al., 2012). Following exposure, C2C12 cells were washed twice with PBS and fixed with 4% paraformaldehyde for 15 min at room temperature. To induce permeabilization, cells were treated with 500 µL of 0.03% TX-100 in PBS for 10 min. As a blocking step, BSA (5% (w/v) in PBS) was added and incubated for 1 h at room temperature. Cells were then incubated overnight with a 1:400 in PBS-diluted mouse anti-mouse IgG primary antibody MyHC I or MyHC II at 4°C, followed by incubation with 1:500 in PBS-diluted secondary antibody, Alexa Fluor 488 goat anti-mouse IgG, for 45 min at room temperature. After MyHC staining, the cell nuclei were stained with 1:2,500 in PBS-diluted DAPI for 15 min at room temperature. A control condition was performed in which the secondary antibody was added without primary antibody. Images were obtained using an EVOS FL Digital Inverted Fluorescence microscope (Thermo Fisher Scientific, Waltham, MA, USA). At least six areas per well were selected that represented an average of the well containing multiple myotubes (≥3 nuclei per cell). For DAPI staining, a blue light cube with excitation at γ=357 and emission at γ=447 was employed, whereas Green Fluorescent Protein (GFP) signals were captured using a green light cube with excitation at γ=470 and emission at γ=525. Image analysis was performed with a custom script (published in Dataverse; see data availability statement) using ImageJ 1.54b software (NIH, Bethesda, MD, USA), and background fluorescence was subtracted prior to quantification. Myotubes were digitally saved as a shape. The center of each nucleus was recognized as a pixel. Cellular shapes not containing ≥3 nucleus pixels were excluded from the analysis. Immunofluorescence staining intensity of the myotubes was measured. Samples were expressed as fold change relative to the control group, which was not exposed to SCFAs.

### 2.5 Statistical analyses

Statistical tests were performed, and graphical illustrations were made using GraphPad Prism 9.4 software (GraphPad, Prism, La Jolla, CA, USA). Normality of the data distribution was checked with the Shapiro–Wilk test. At each time point, individual treatment conditions were compared with the corresponding control. A one-way analysis of variance (ANOVA) was performed. When ANOVA indicated significance, comparisons between treatment groups and control were conducted using Dunnett’s multiple comparisons test. At least three independent experiments were performed in duplicates. P-values <0.05 were considered statistically significant. All values are presented as the mean ± standard error of mean (SEM). N is the number of independent experiments, whereas n reflects the number of replicates.

## 3 Results

### 3.1 A mixture of SCFAs upregulated Pgc1α and Tfam gene expression at 8 and 24 h

**Figure 2:**
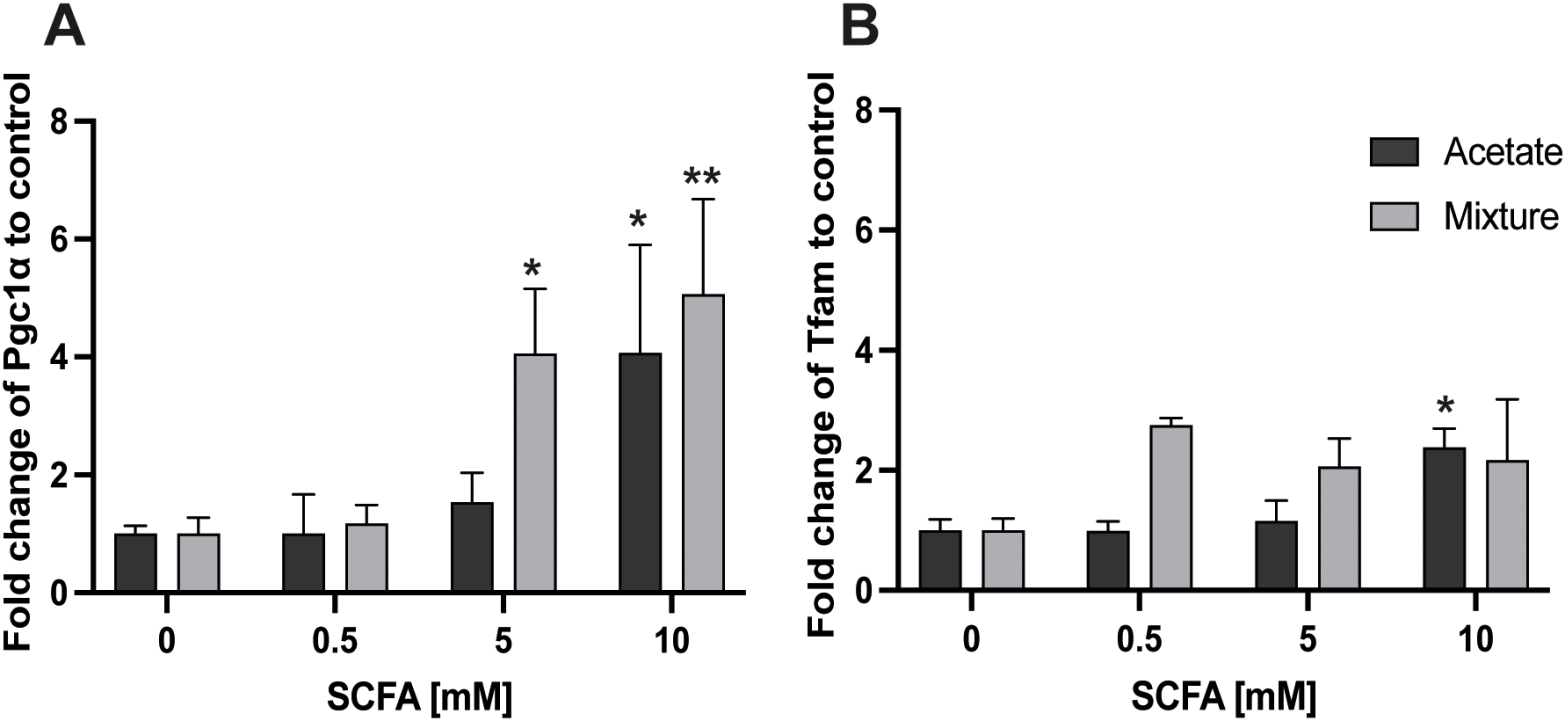
Gene expression response of *Pgc1α* and *Tfam* in C2C12 myotubes after exposure to acetate and a mixture of SCFAs for 8 (h) Acetate at 10 mM and the mixture at 5 and 10 mM increased *Pgc1α* gene expression in C2C12 myotubes after 8 h of exposure **(A)**. *Tfam* gene expression was increased by acetate at 10 mM, but was not changed by a mixture of SCFAs **(B)** (N≥3; n=3). A one-way ANOVA followed by Dunnett’s multiple comparisons test was performed to compare each treatment condition with the control (0 mM SCFA exposure). All data are presented as mean ± SEM. *p < 0.05, **p < 0.01, compared with control and relative to the housekeeping genes (GAPDH, RPL13A, and YWHAZ) according to the Pfaffl method.

Eight-hour exposure of C2C12 myotubes to 10 mM acetate increased the *Pgc1α* gene expression by 4.07 ± 1.84-fold compared with control (*p* < 0.05) (**Figure 2A**). Similarly, a mixture of SCFAs at total concentrations of 5 and 10 mM significantly increased *Pgc1α* gene expression compared with the control condition to 4.05 ± 1.11 and 5.06 ± 1.61, respectively (*p* < 0.05 and *p* < 0.01) (**Figure 2A**). Acetate at 10 mM increased *Tfam* gene expression in C2C12 at 8 h 2.38 ± 0.31-fold (*p* < 0.05) (**Figure 2B**), whereas the mixture of SCFAs did not show a significant change. In contrast, 8-h exposure of acetate as well as a mixture of SCFAs did not change the gene expression of *Nrf1* or *Nrf2a* compared with control in C2C12 myotubes (*p* > 0.05) (**Supplementary Figures 1A, B**).

**Figure 3:**
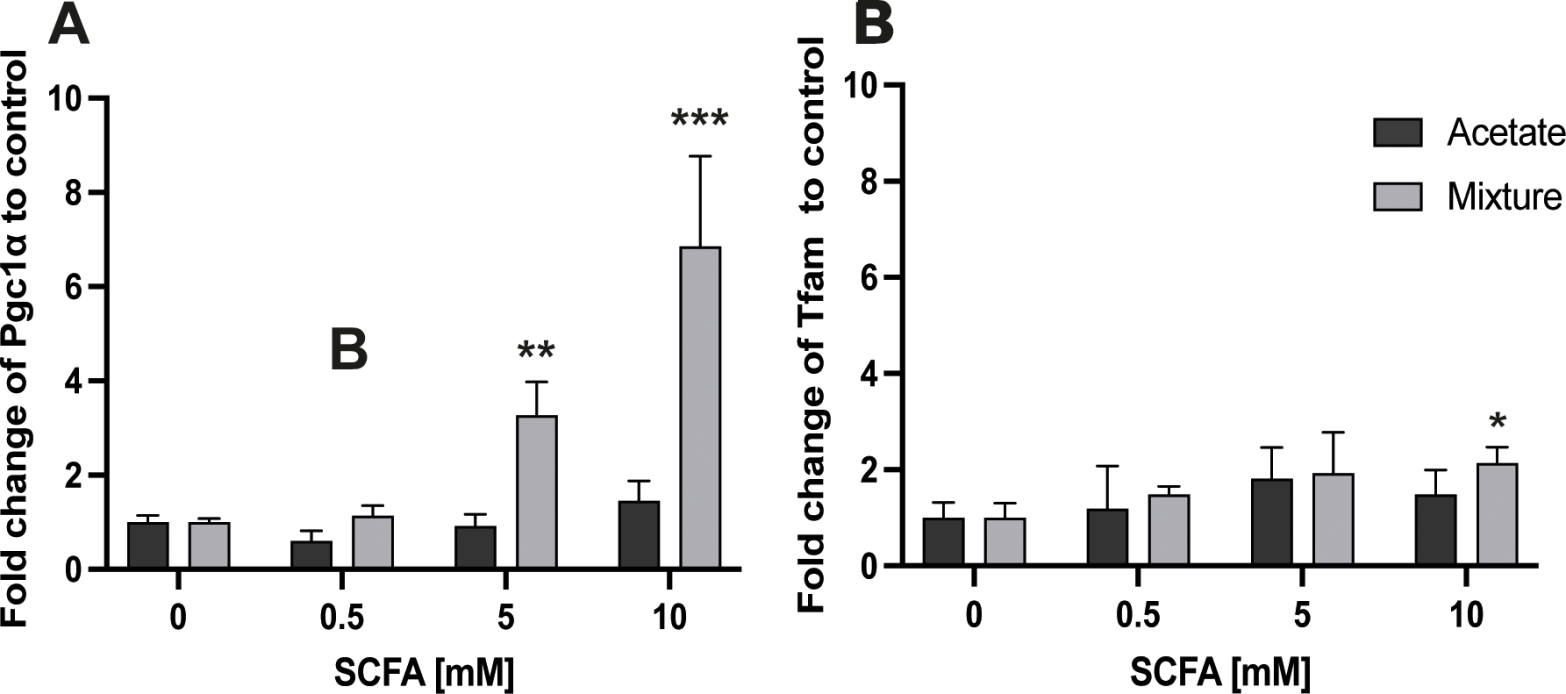
Gene expression response of *Pgc1α* and *Tfam* in C2C12 myotubes after exposure to acetate and a mixture of SCFAs for 24 (h) Exposure of C2C12 myotubes for 24 h to acetate did not change *Pgc1α* gene expression, whereas the mixture of SCFAs at 5 and 10 mM increased *Pgc1α* gene expression **(A)**. Exposure of C2C12 myotubes to 10 mM of the mixture of SCFAs increased the *Tfam* gene expression compared with 0 mM **(B)** (N = 3; n=3). A one-way ANOVA followed by Dunnett’s multiple comparisons test was performed to compare each treatment condition with the control (0 mM SCFA exposure). All data are presented as mean ± SEM. *p < 0.05, **p < 0.01, ***p < 0.001 compared with control and relative to the housekeeping genes (GAPDH, RPL13A, and YWHAZ) according to the Pfaffl method.

No change in *Pgc1α* gene expression was seen after exposing C2C12 myotubes to acetate for 24 h (*p* > 0.05) (**Figure 3A**). In contrast to acetate alone, 24-h exposure of C2C12 myotubes to 5 and 10 mM of the SCFA mixture resulted in an increase in *Pgc1α* gene expression with a fold change of 3.28 ± 0.70 and 6.85 ± 1.91 compared with control, respectively (*p* < 0.01 and *p* < 0.001; respectively) (**Figure 3A**). No changes in *Tfam* gene expression were observed in C2C12 myotubes after 24-h exposure to acetate (*p* > 0.05) (**Figure 3B**). *Tfam* gene expression was 2.14 ± 0.33-fold higher compared with control after 24-h exposure of C2C12 myotubes to 10 mM of the SCFA mixture (*p* < 0.05) (**Figure 3B**). Gene expression of *Nrf1* and *Nrf2a* remained unchanged after 24-h exposure to either acetate or the SCFA mixture (*p* > 0.05) (**Supplementary Figures 2A, B**).

### 3.2 Eight-hour exposure to acetate or 10 mM of the mixture increased Pparα in C2C12 myotubes

**Figure 4:**
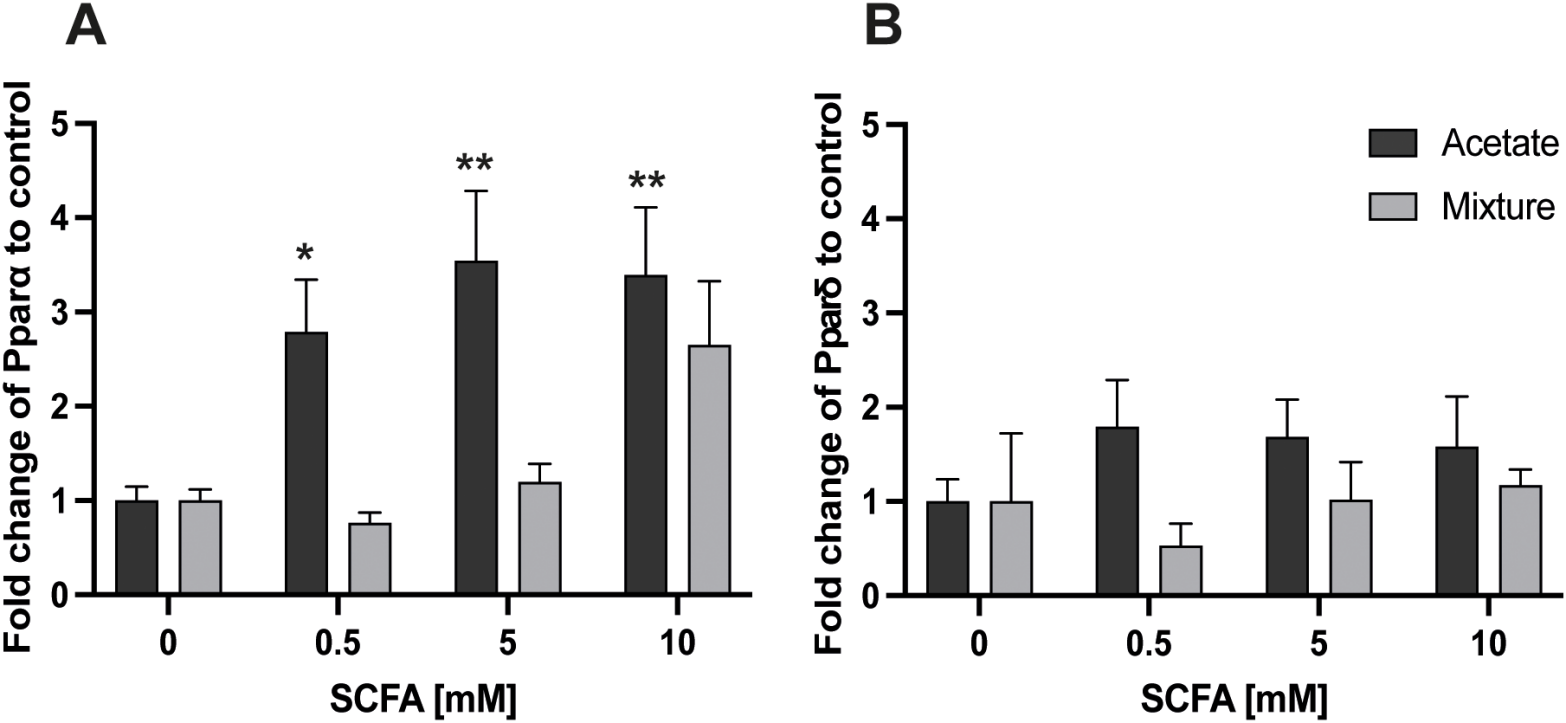
Gene expression responses of *Pparα* and *Pparδ* in C2C12 myotubes after exposure to acetate and a mixture of SCFAs for 8 (h) Acetate at 0.5, 5, and 10 mM increased the *Pparα* gene expression in C2C12 myotubes after 8 h of exposure **(A)**. Both acetate and the mixture did not alter the gene expression of *Pparδ***(B)** (N = 3; n=3). A one-way ANOVA followed by Dunnett’s multiple comparisons test was performed to compare each treatment condition with the control (0 mM SCFA exposure). All data are presented as mean ± SEM. *p < 0.05, **p < 0.01, ***p < 0.001 compared with control and relative to the housekeeping genes (GAPDH, RPL13A, and YWHAZ) according to the Pfaffl method.

Acetate increased *Pparα* gene expression, with a fold change of 2.79 ± 0.55, 3.54 ± 0.74, and 3.39 ± 0.71 in C2C12 myotubes after 8-h exposure to 0.5, 5, and 10 mM, respectively, compared with control (*p* < 0.05, *p* < 0.01, *p* < 0.01; respectively) (**Figure 4A**). In contrast, no significant changes in *Pparα* gene expression were observed after 8-h exposure to the SCFA mixture at any concentration (p > 0.05) (**Figure 4A**). No significant alterations in *Ppar*δ gene expression were observed after 8-h exposure to acetate or the mixture compared with control (*p* > 0.05) (**Figure 4B**).

**Figure 5:**
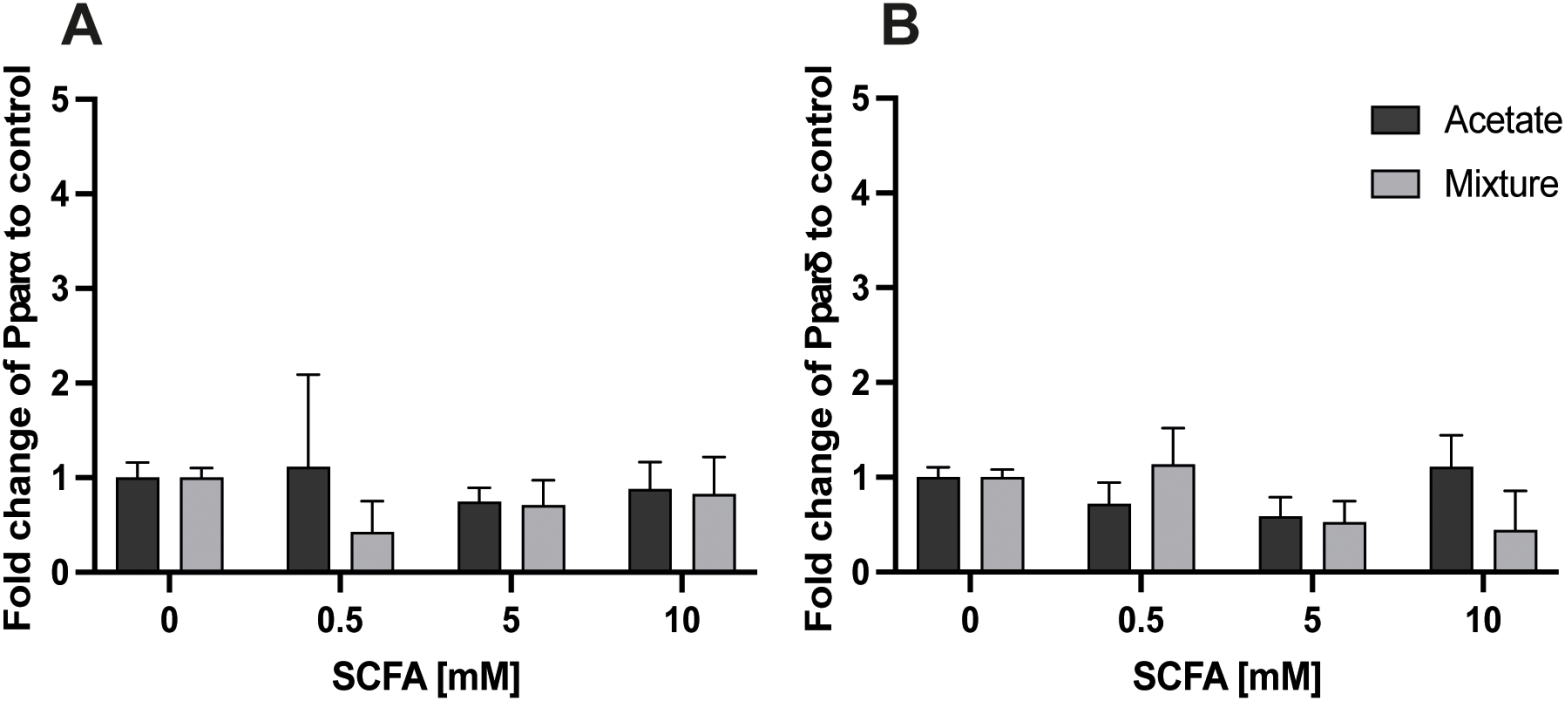
Gene expression response of *Pparα* and *Pparδ* in C2C12 myotubes after exposure to acetate and a mixture of SCFAs for 24 (h) Both 24-h exposure of C2C12 myotubes to acetate and the mixture did not alter gene expression of *Pparα***(A)** and *Pparδ***(B)** compared with control (N = 3; n=3). A one-way ANOVA followed by Dunnett’s multiple comparisons test was performed to compare each treatment condition with the control (0 mM SCFA exposure). All data are presented as mean ± SEM and relative to the housekeeping genes (GAPDH, RPL13A, and YWHAZ) according to the Pfaffl method.

At 24 h, no significant changes were observed in the gene expression levels of *Pparα* and *Pparδ* after exposure to acetate nor the SCFA mixture compared with control (*p* > 0.05) (**Figures 5A, B**).

### 3.3 Acetate and a mixture of SCFAs increased Myh7 gene expression in C2C12 myotubes after 24-h exposure

**Figure 6:**
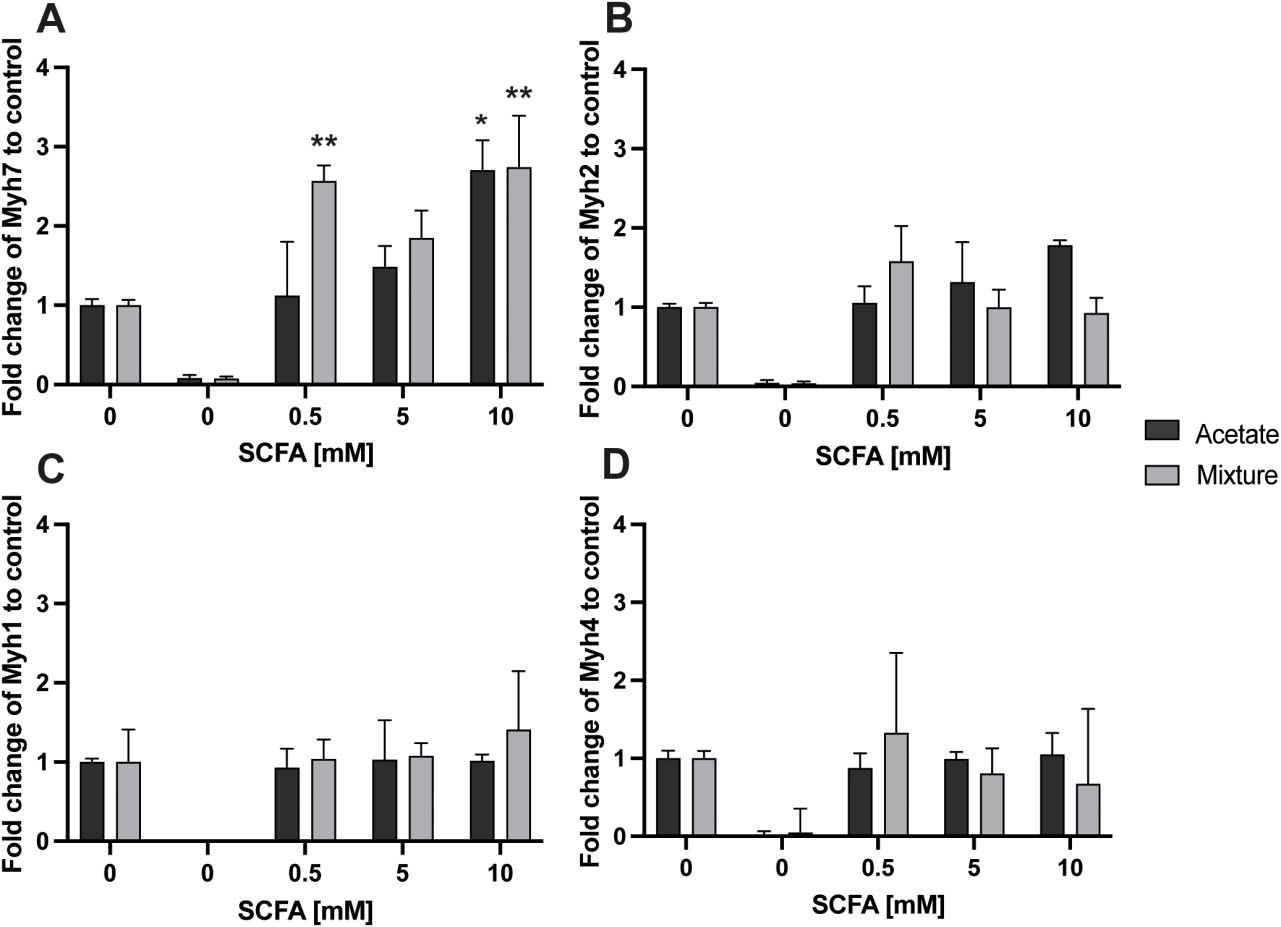
Gene expression response of *Myh7*, *Myh2*, *Myh1*, and *Myh4* in C2C12 myotubes after exposure to acetate and a mixture of SCFAs for 24 (h) Acetate at 10 mM and the mixture at 0.5 and 10 mM increased *Myh7* gene expression compared with 0 mM SCFA **(A)**. Acetate and the mixture exposure did not change *Myh2***(B)**, *Myh1***(C)**, and *Myh4***(D)** gene expression after 24 h (N = 3; n=2). A one-way ANOVA followed by Dunnett’s multiple comparisons test was performed to compare each treatment condition with the control (0 mM SCFA exposure). All data are presented as mean ± SEM. *p < 0.05, **p < 0.01, ***p < 0.001 compared with control and relative to the housekeeping genes (GAPDH, RPL13A, and YWHAZ) according to the Pfaffl method.

Myoblasts, used as a negative control, showed low levels of Myh gene expression compared with control (*p* < 0.001) (**Figure 6**). At the 8-h time point, no significant changes in *Myh7*, *Myh2*, *Myh1*, and *Myh4* gene expression were observed in myotubes across the tested conditions (*p* > 0.05) (data not shown). Exposure to acetate at 10 mM for 24 h increased the gene expression of *Myh7* by 2.76 ± 0.33-fold (*p* < 0.05), compared with control (**Figure 6A**). After 24 h of exposure to acetate, no other differences in gene expression in C2C12 myotubes were observed (*p* > 0.05) (**Figures 6B–D**). At the 24-h time point, exposure to the SCFA mixture at concentrations of 0.5 and 10 mM increased *Myh7* gene expression by 2.57 ± 0.20- and 3.13 ± 0.57-fold, respectively, compared with control (*p* < 0.01) (**Figure 6A**). The gene expression levels of *Myh2*, *Myh1*, and *Myh4* were not significantly different from control after 24-h exposure to the SCFA mixture (*p* > 0.05) (**Figures 6B–D**).

### 3.4 Acetate increased MyHC I fold change in C2C12 myotubes after 48-h exposure

**Figure 7:**
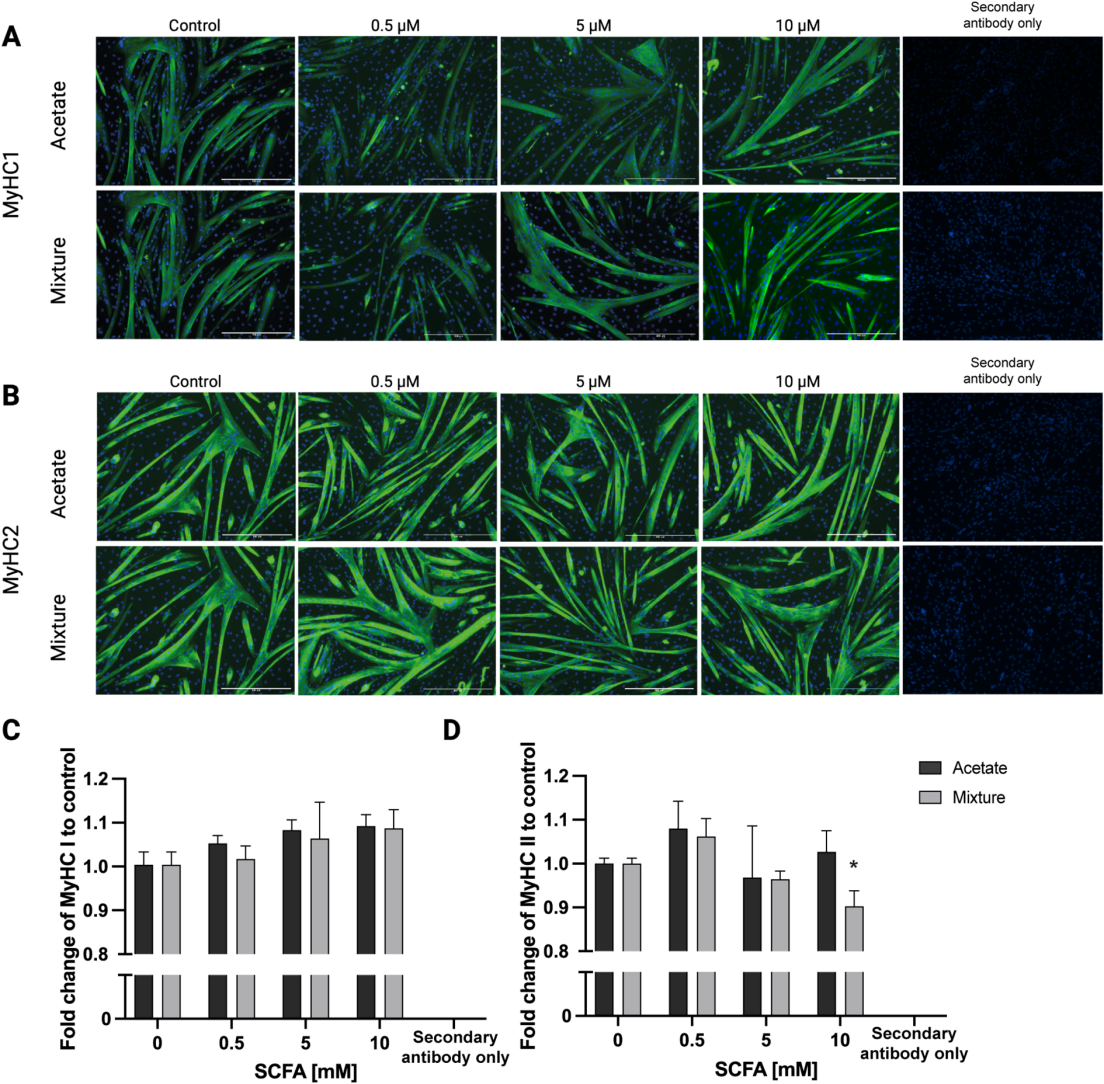
MyHC I and MyHC II fold change in C2C12 myotubes after exposure to acetate and a mixture of SCFAs for 48 h. Representative immunofluorescence images of MyHC I and MyHC II in C2C12 myotubes after 48 h exposure to acetate or a mixture of SCFAs, to support the quantitative analyses **(A, B)**. A 48-h exposure of C2C12 myotubes to acetate or the SCFA mixture did not change MyHC I fold change **(A, C)**. Acetate did not change MyHC II fold change in C2C12 myotubes after 48-h exposure, whereas 10-mM exposure to the mixture decreased MyHC II fold change in C2C12 myotubes compared with control **(B, D)**. A control condition was performed in which the secondary antibody was added without a primary antibody (secondary antibody only). (N = 3). A one-way ANOVA followed by Dunnett’s multiple comparisons test was performed to compare each treatment condition with the control (0 mM SCFA exposure). All data are presented as mean ± SEM. *p < 0.05, compared with control.

First, the effects of acetate and second the effects of the mixture on MyHC I fold change were indicated. 48 h of exposure of C2C12 myotubes to acetate did not significantly change MyHC I or MyHC II fold change at any concentration (*p* > 0.05) compared with control (**Figures 7A, C**). Exposure to the SCFA mixture did not change MyHC I fold change compared with control (*p* > 0.05) (**Figure 7C**), whereas 10 mM of the mixture decreased MyHC II fold change by 0.90 ± 0.04-fold compared with control (*p* < 0.05) (**Figures 7B, D**).

**Figure 8:**
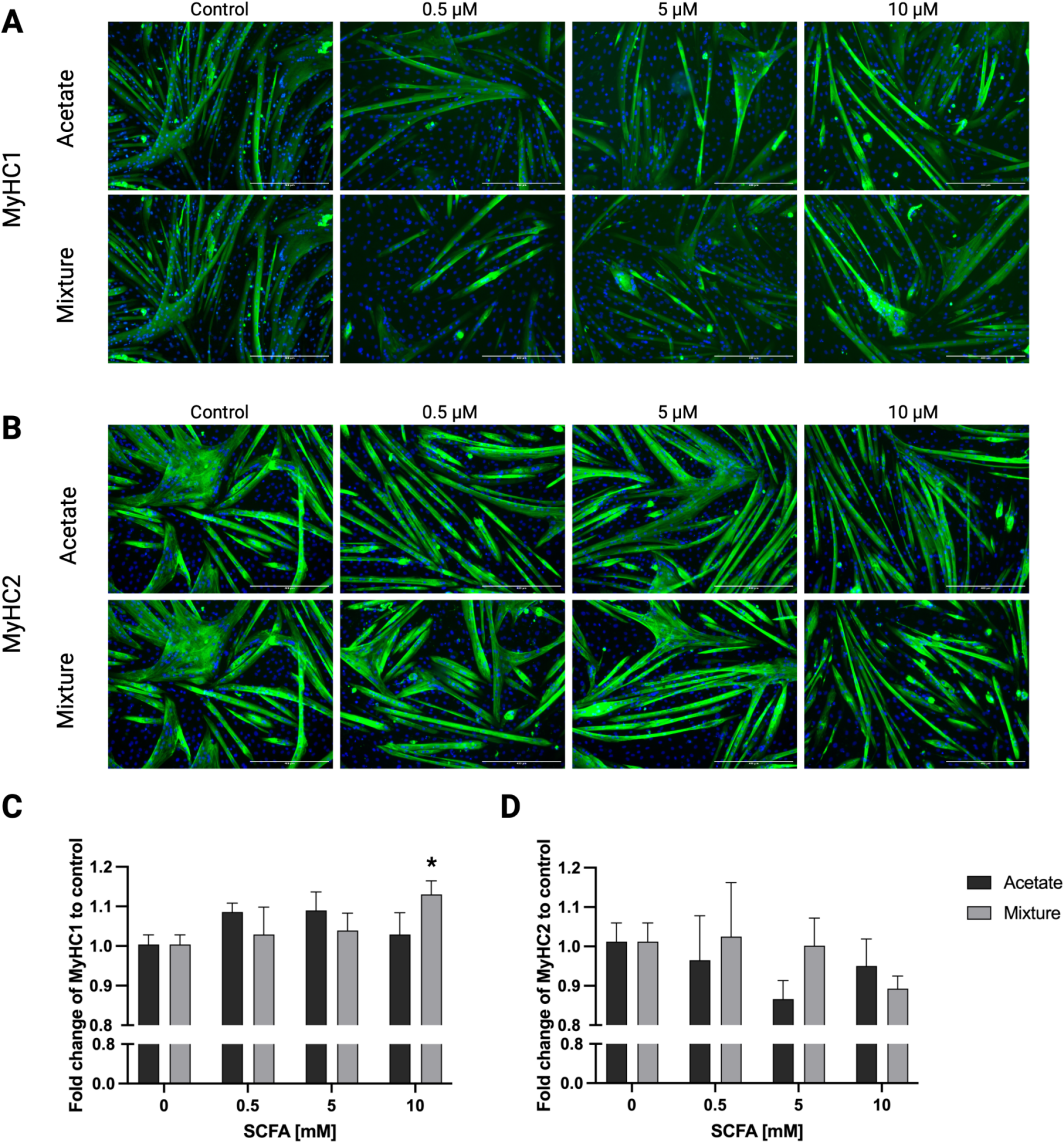
MyHC I and MyHC II fold change compared with 0-mM SCFA exposure in C2C12 myotubes after exposure to acetate and a mixture of SCFAs for 72 h. Representative immunofluorescence images of MyHC I and MyHC II in C2C12 myotubes after 72 h exposure to acetate or a mixture of SCFAs, supporting the quantitative analyses **(A, B)**. Exposure of C2C12 myotubes for 72 h to acetate did not change MyHC I fold change, whereas 10 mM of the mixture of SCFAs increased MyHC I fold change compared with control **(A, C)**. Acetate and the mixture of SCFAs did not change MyHC II fold change in C2C12 myotubes **(B, D)**. (N = 3). A one-way ANOVA followed by Dunnett’s multiple comparisons test was performed to compare each treatment condition with the control (0 mM SCFA exposure). All data are presented as mean ± SEM. *p < 0.05, compared with control.

Again, the effects of acetate on MyHC II fold change were shown followed by effects of the mixture on this parameter. Exposing C2C12 myotubes to acetate for 72 h did not change MyHC I fold change compared with control (*p* > 0.05) (**Figures 8A, C**). In contrast, exposing C2C12 myotubes to 10 mM of the SCFA mixture increased MyHC I fold change compared with control by 1.13 ± 0.03-fold (*p* < 0.05) (**Figure 8C**). Exposure of C2C12 myotubes to acetate or the mixture did not induce a significant change in MyHC II fold change (**Figures 8B, D**).

## 4 Discussion

SCFAs have been implicated in influencing muscle phenotype, yet their precise effects remain to be investigated. In this study, we investigated the potential of SCFAs, particularly acetate and an SCFA mixture in a physiologically relevant amount of acetate:propionate:butyrate, i.e., 60:20:20% respectively, to modify the oxidative muscle phenotype of C2C12 myotubes. We observed a significant upregulation of genes associated with mitochondrial biogenesis and fatty acid metabolism, including *Pgc1α*, *Tfam*, and *Pparα*, following exposure to both acetate and the SCFA mixture. This effect was also shown for *Myh7* gene expression. Interestingly, the SCFA mixture exhibited this effect at lower concentrations compared with acetate alone except for *Pparα*. Immunofluorescence staining further demonstrated alterations in muscle protein composition, with an increase in MyHC I and a decrease in MyHC II levels in response to SCFA exposure.

Skeletal muscle has the ability to adapt to various physiological stimuli, including dynamic shifts in fiber types from slow to fast skeletal muscle type and vice versa (Schiaffino and Reggiani, 2011; Plotkin et al., 2021). In this study, myotubes exposed to acetate and the SCFA mixture for 24 h exhibited an increase in *Myh7* gene expression. Immunofluorescence staining showed enhanced MyHC I protein levels after 72 h of exposure to the SCFA mixture. Interestingly, at a concentration of 10 mM, the SCFA mixture reduced MyHC II protein levels in myotubes after 48 h, suggesting a shift from fast to slow muscle fiber characteristics. Despite the observed decrease in MyHC II levels by immunofluorescence staining, no corresponding decrease was detected at the gene expression level. However, this study was performed in the mouse C2C12 cell line. In mice, there are four fiber types, and more than in humans, MyHC IIa is highly oxidative (Gouspillou et al., 2014). It is important to mention that the antibody used in our study recognized both MyHC IIa (fast-twitch oxidative) and MyHC IIx (fast-twitch glycolytic) isoforms and thus may have masked any shifts between these fiber types (Talbot and Maves, 2016). Previously, the Myod family inhibitor (Mdfi) was found to promote the conversion of fast-to-slow-twitch muscle phenotype in C2C12 myotubes with increased MyHC I and MyHC IIa and reduced MyHC IIx (Huang et al., 2021).

The increase in *Myh7* gene expression and MyHC I immunofluorescence staining, alongside the observed reduction in MyHC II, despite the absence of a corresponding decrease in *Myh1*, *Myh2*, and *Myh4* gene expression, suggests the involvement of a posttranslational mechanism. The shift toward an oxidative phenotype is typically associated with increased mitochondrial content and metabolic adaptations. While oxidative fibers often exhibit altered redox balance, this study did not directly measure oxidative stress markers, and future research should assess whether SCFA-induced fiber shifts are associated with changes in mitochondrial ROS regulation (Anderson and Neufer, 2006; Picard et al., 2011; Picard et al., 2012).

In addition to the changes in MyHC isoforms, several other factors play a role in determining muscle fiber characteristics, including metabolic attributes (Schiaffino and Reggiani, 2011). In this study, 8-h exposure to a mixture of SCFA at concentrations of 5 and 10 mM, along with acetate at 10 mM, resulted in an elevation of *Pgc1α* levels. Only the mixture demonstrated a continuous upregulation in both *Pgc1α* and *Tfam* expression at 24 h, emphasizing its prolonged impact on mitochondrial biogenesis. The oxidative phenotype of muscle, which is crucial for endurance exercise performance, is associated with mitochondria content and activity and with fatty acid oxidation capacity. Previous studies have shown that shifts toward an oxidative muscle fiber phenotype and increased expression of mitochondrial biogenesis–related genes are associated with functional improvements in mitochondrial content and respiratory capacity (Lin et al., 2002; Mishra et al., 2015; Chen et al., 2020; Dong and Tsai, 2023). One key player in this regulatory network is the PGC1α pathway (Kang and Li Ji, 2012). PGC1α serves as a master regulator of mitochondrial biogenesis and oxidative metabolism and is known to facilitate the transformation of glycolytic fibers into oxidative fibers by enhancing mitochondrial biogenesis (Lin et al., 2002). Slow-twitch muscle fibers have increased levels of Pgc1α compared with fast-twitch muscle fibers and are upregulated by endurance exercise in both rodents and humans (Liang and Ward, 2006). Pgc1α stimulates the expression of *Nrf-1 and -2*, which regulate the transcription of numerous mitochondrial genes such as *Tfam* (26). *Tfam* encodes a mitochondrial protein that is essential for the replication and transcription of mitochondrial DNA. Mice overexpressing Pgc1α exhibit increased mitochondrial content, higher oxidative enzyme levels in fast muscle fibers, and a greater proportion of oxidative type I and IIa fibers, enhancing resistance to fatigue in comparison with wild-type mice (Miura et al., 2003). Comparable with findings from this study, 24-h exposure of 10 mM of the total SCFA mix in a relative amount of 60:25:15% of acetate:propionate:butyrate increased the *Pgc1α* expression in C2C12 myotubes (Lahiri et al., 2019). Simultaneous to the increase in *Pgc1α* gene expression, exposure to the different concentrations of the SCFA mixture for 8 h resulted in an increase in *Tfam* expression, whereas acetate only increased *Tfam* expression at 10 mM. Gene expression levels of *Tfam* remained elevated at 10 mM exposure of the mixture after 24 h. Interestingly, in contrast to *Tfam* and *Pgc1α* regulation, only acetate exposure increased *Pparα* expression at all concentrations. These observations suggest that SCFAs may exert their effects on muscle fiber type through a multifaceted mechanism involving the interplay of *Pgc1α* and *Tfam*, thereby affecting mitochondrial biogenesis and ultimately contributing to the transition from fast to slow muscle fiber type.

SCFAs exert a variety of different effects on skeletal muscle, engaging distinct mechanisms of action (Giron et al., 2022). Their uptake by skeletal muscle can occur through both active and passive diffusion processes, unlike LCFAs such as PUFAs and palmitate, which require specific transporters such as CD36 and FATP for cellular uptake. Additionally, SCFAs can activate GPR41 and GPR43 receptors in skeletal muscle cells, initiating signaling cascades that impact metabolic and inflammatory responses. GPR43 displays a preference for shorter-chain fatty acids, specifically acetate and propionate, whereas GPR41 exhibits a higher binding affinity for, butyrate, and propionate compared with acetate (Sivaprakasam et al., 2016). For instance, activation of GPR41 enhanced energy expenditure and insulin sensitivity, contributing to improved glucose uptake in C2C12 myotubes (Lee et al., 2023). GPR43 in L6 skeletal muscles triggered the upregulation of gene expressions linked to slow-twitch fiber characteristics (Maruta and Yamashita, 2020). The observed activation of *Pgc1α* and *Tfam* by the SCFA mixture at lower concentrations compared with acetate suggests a potential involvement of GPR41, possibly influenced by butyrate and propionate in promoting genes related to mitochondrial biogenesis. GPR43, potentially associated with acetate, may play a role in activating *Pparα*.

*PPARα* and *PPARδ* have been implicated as key regulators of lipid and glucose metabolism and muscle fiber type (Finck et al., 2005; Ehrenborg and Krook, 2009). Previous studies have shown that LCFAs, such as PUFAs and palmitate, influence muscle fiber type composition by enhancing oxidative capacity through PPAR signaling and mitochondrial biogenesis (de Wilde et al., 2008; Mizunoya et al., 2013). Our findings suggest that SCFAs may exert similar effects, although potentially through distinct mechanisms. Interestingly, acetate increased *Pparα* gene expression at ≥0.5mM, whereas the SCFA mixture showed no effect, with no significant effect observed on *Pparδ* gene expression. Previous research has shown that acetate treatment increased *Pparα* expression in rabbit muscle (Liu et al., 2019). Similarly, Hong et al. reported a significant increase in skeletal muscle *Pparα* mRNA expression in mice supplemented with butyrate for 10 days, although no corresponding effect was observed at the protein level (Hong et al., 2016). PPARδ, predominantly found in skeletal muscle, plays a crucial role in lipid and glucose metabolism and muscle fiber type regulation (36). In contrast to our findings, which showed no change in *Pparδ* gene expression following SCFA mixture exposure, butyrate alone enhanced *Pparδ* gene expression in both L6 myotubes and skeletal muscle of C57BL/6J mice *in vivo* (Gao et al., 2009). However, unlike SCFAs, PUFAs have been shown to more strongly activate *PPARδ*, which plays a role in shifting muscle fiber composition toward an oxidative phenotype. Future investigations using specific GPR inhibitors combined with single SCFA exposures and varied SCFA/PUFA mix ratios could help unravel their specific roles in modulating muscle fiber phenotypes.

The C2C12 myotube model is a widely used and generally accepted *in vitro* system to study muscle physiology, including the exploration of muscle fiber types (Denes et al., 2019). These cells provide a controlled environment to investigate gene and protein expression changes in response to various stimuli. However, while the C2C12 model presents numerous advantages, it is essential to acknowledge its inherent limitations when investigating muscle fiber types. A key limitation is that C2C12 myotubes do not fully recapitulate the characteristics of mature adult muscle fibers (Abdelmoez et al., 2020). As C2C12 cells originate from a mouse model, the muscle fiber type composition of these cells may not fully mirror the intricacies of human muscle physiology (Schiaffino and Reggiani, 2011). Unlike in humans, mice exert four different muscle fiber types, and in contrast to humans, MyHC IIa exhibits the highest mitochondrial content in mouse muscle. Moreover, the differentiation process undergone by C2C12 cells may not entirely capture the multifaceted diversity and complexity inherent to muscle fiber types in an *in vivo* setting. Alternative models such as primary human myoblasts or engineered culture systems may offer more mature myotubes with characteristics closer to adult muscle (Rando and Blau, 1994; Brunetti et al., 2021). Additionally, while this study provides valuable insights into the gene expression and protein localization of MyHC I and II in C2C12 myotubes in response to SCFAs, the lack of direct protein quantification through techniques such as Western blotting or ELISA is considered a limitation. Although gene expression and immunofluorescence analysis offer complementary insights into protein dynamics, further validation of these findings at the protein level could strengthen our conclusions.

In conclusion, while both acetate and combinations of SCFAs modulate the expression of genes related to oxidative muscle fiber types, the SCFA mixture demonstrates a more pronounced effect at lower concentrations, suggesting a potential enhancement of the shift toward oxidative muscle characteristics compared with acetate alone. Future research should focus on understanding the intricate mechanisms related to GPR41 and 43 binding underlying the impact of SCFAs on muscle fiber type conversion and enhanced oxidative capacity. Further validation in *in vivo* models is warranted to confirm the physiological relevance of these outcomes. Exploring strategies such as pre- or probiotics to modulate the gut microbiota could stimulate a more diverse SCFA profile leading to a ratio of SCFA, as used in this study. This may modulate the muscle fiber type and oxidative capacity, which could be interesting for enhancing muscle health in clinical conditions and among endurance athletes.

## Data Availability

Raw numerical data and complete summaries of statistics for **Figure 2**-**8** are available in Dataverse https://doi.org/10.34894/ZYZGPE. Additional results that support the findings of this study are also available from the corresponding author upon reasonable request.

## Supplementary material

Supplementary Figure 1 Gene expression of *Nrf1* and *Nrf2a* in C2C12 myotubes after 8 h exposure to acetate and a mixture of SCFAs. Exposure to acetate or a mixture of SCFAs for 8 h did not alter gene expression of *Nrf1***(A)** or *Nrf2a***(B)** in C2C12 myotubes (N ≥ 3; n = 3). All data are presented as mean ± SEM and relative to the housekeeping genes (GAPDH, RPL13A, and YWHAZ) according to the Pfaffl method.

Supplementary Figure 2 Gene expression of *Nrf1* and *Nrf2a* in C2C12 myotubes after 24 h exposure to acetate and a mixture of SCFAs. Exposure to acetate or a mixture of SCFAs for 24 h did not alter gene expression of Nrf1 **(A)** or Nrf2a **(B)** in C2C12 myotubes (N = 3; n = 3). All data are presented as mean ± SEM and normalized to GAPDH, RPL13A, and YWHAZ using the Pfaffl method.
