# Supplementary figures and images for: Acetate and combinations of short-chain fatty acids increase oxidative phenotype and contribute to muscle fiber type shift in myotubes
Source: Front Physiol. 2026 Mar 25;17:1757576. doi: 10.3389/fphys.2026.1757576 (PMC13056642; doi:10.3389/fphys.2026.1757576)

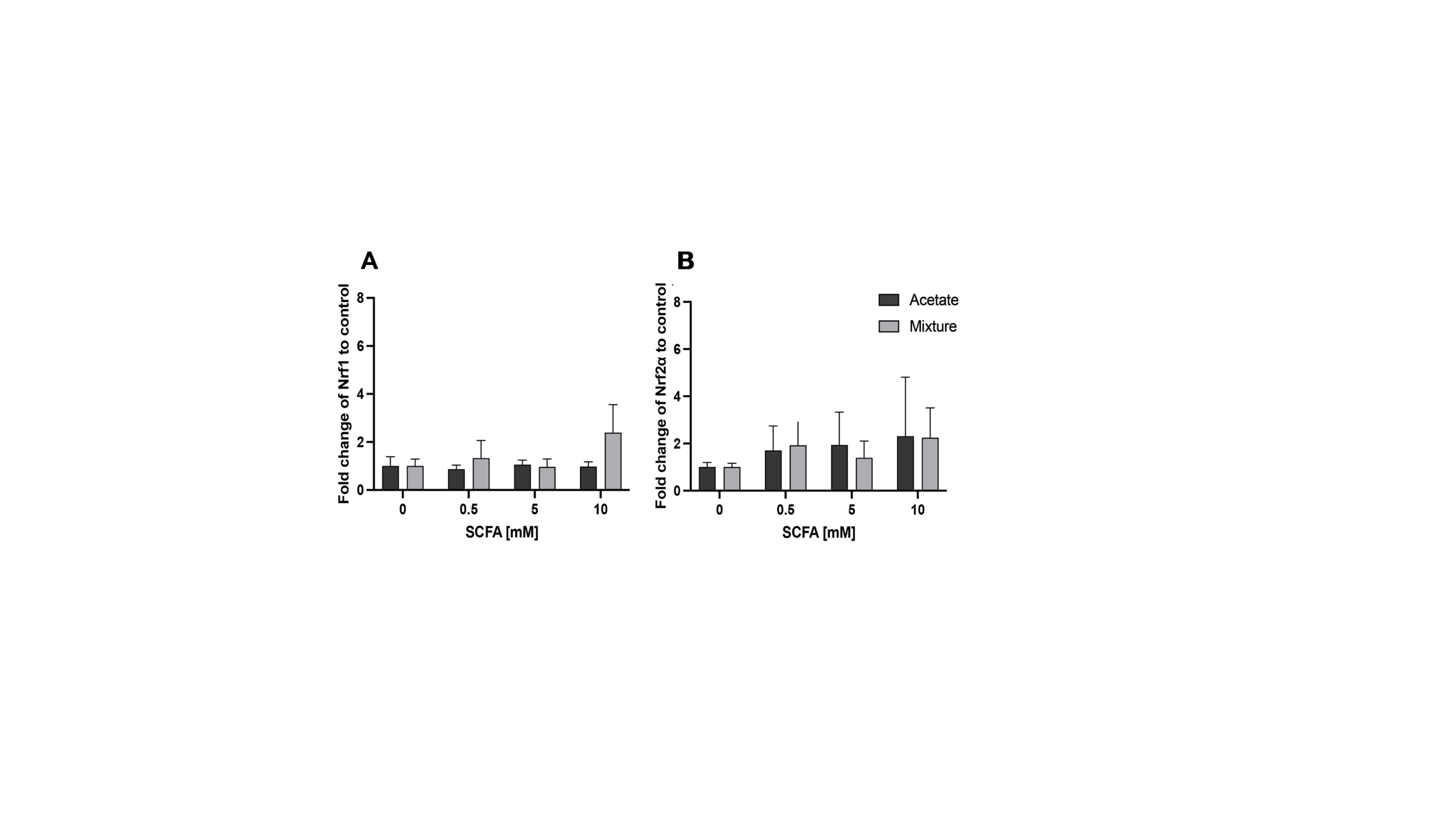

Supplement: Supplementary Figure 1 — Gene expression of Nrf1 and Nrf2a in C2C12 myotubes after 8 h exposure to acetate and a mixture of SCFAs. Exposure to acetate or a mixture of SCFAs for 8 h did not alter gene expression of Nrf1(A) or Nrf2a(B) in C2C12 myotubes (N ≥ 3; n = 3). All data are presented as mean ± SEM and relative to the housekeeping genes (GAPDH, RPL13A, and YWHAZ) according to the Pfaffl method. [file Image1.jpeg]

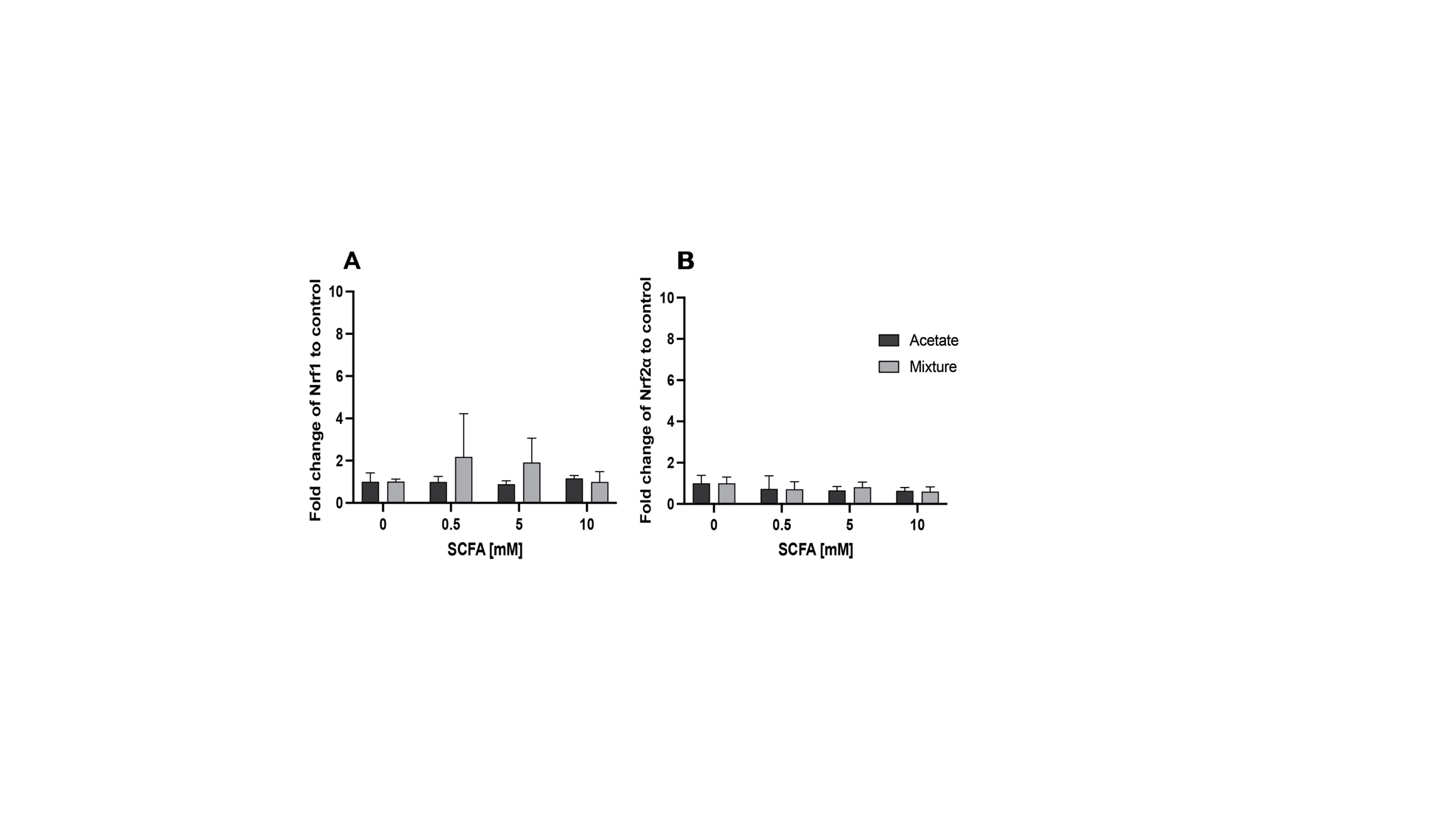

Supplement: Supplementary Figure 2 — Gene expression of Nrf1 and Nrf2a in C2C12 myotubes after 24 h exposure to acetate and a mixture of SCFAs. Exposure to acetate or a mixture of SCFAs for 24 h did not alter gene expression of Nrf1 (A) or Nrf2a (B) in C2C12 myotubes (N = 3; n = 3). All data are presented as mean ± SEM and normalized to GAPDH, RPL13A, and YWHAZ using the Pfaffl method. [file Image2.jpeg]
